# Exploring the Release of Elastin Peptides Generated from Enzymatic Hydrolysis of Bovine Elastin via Peptide Mapping

**DOI:** 10.3390/molecules28227534

**Published:** 2023-11-10

**Authors:** Jianan Zhang, Yang Liu, Liwen Jiang, Tiantian Zhao, Guowan Su, Mouming Zhao

**Affiliations:** 1 School of Food Science and Engineering, South China University of Technology, Guangzhou 510640, China; fez.jianan@foxmail.com (J.Z.); fegwsu@scut.edu.cn (G.S.); 2 Guangdong Food Green Processing and Nutrition Regulation Technologies Research Center, Guangzhou 510650, China; 3 College of Food Science and Technology, Hunan Agricultural University, Changsha 410125, China; hnndjlw@163.com; 4 Sericulture & Agri-Food Research Institute, Guangdong Academy of Agricultural Sciences, Key Laboratory of Functional Foods, Ministry of Agriculture and Rural Affairs, Guangdong Key Laboratory of Agricultural Products Processing, Guangzhou 510610, China; fettzhao1989@163.com

**Keywords:** elastin peptides, 2D LC-MS, peptide fingerprinting, enzymatic cleavage patterns, elastase inhibitory activity

## Abstract

To enhance the understanding of enzymatic hydrolysis and to accelerate the discovery of key bioactive peptides within enzymatic products, this research focused on elastin as the substrate and investigated the variations in peptide profiles and the production of key bioactive peptides (those exceeding 5% of the total) and their impacts on the biological activity of the hydrolysates. Through the application of advanced analytical techniques, such as stop-flow two-dimensional liquid chromatography and ultra-high-performance liquid chromatography-tandem mass spectrometry, the research tracks the release and profiles of peptides within elastin hydrolysates (EHs). Despite uniform peptide compositions, significant disparities in peptide concentrations were detected across the hydrolysates, hinting at varying levels of bioactive efficacy. A comprehensive identification process pinpointed 403 peptides within the EHs, with 18 peptides surpassing 5% in theoretical maximum content, signaling their crucial role in the hydrolysate’s bioactivity. Of particular interest, certain peptides containing sequences of alanine, valine, and glycine were released in higher quantities, suggesting Alcalase^®^ 2.4L’s preference for these residues. The analysis not only confirms the peptides’ dose-responsive elastase inhibitory potential but also underscores the nuanced interplay between peptide content, biological function, and their collective synergy. The study sets the stage for future research aimed at refining enzymatic treatments to fully exploit the bioactive properties of elastin.

## 1. Introduction

Bioactive peptides, composed of 2–50 amino acids, exhibit various physiological properties, including antioxidative, antihypertensive, and antihyperuricemia effects [1,2]. Some of these peptides maintain their physiological effects in vivo when absorbed in their native structures [3]. Consequently, they have emerged as a central subject of modern scientific research and are recognized as functional food ingredients that confer substantial health advantages.

Bioactive peptides derived from food can be integrated into a variety of foods and beverages as health-enhancing constituents [4]. They also hold promise as health foods or dietary supplements, underscoring the vast market demand [5]. They are predominantly procured through the enzymatic hydrolysis of protein-abundant food materials [6]. This hydrolysis, however, produces a myriad of products, with peptide profiles that differ based on enzyme specificity and hydrolytic conditions [7]. Such variations can directly influence the bioactivity of the resulting hydrolysates. Thus, optimizing the bioactivity of these hydrolysis products necessitates a comprehensive grasp of the enzymatic hydrolysis dynamics, spanning from amino acid and peptide release to enzymatic cleavage determinants. A nuanced understanding of the bioactive properties of the components, especially their synergistic effect, is also essential.

Two primary strategies dominate bioactive peptide research. The first necessitates multiple rounds of purification, concentration, and isolation to pinpoint peptides with defined compositions and sequences [8]. Thousands of such peptides are cataloged in the Biopep database [9]. However, this method is labor-intensive and exposes the process to contamination risks and potential peptide degradation [10]. The second strategy harnesses computational simulation, grounded in known bioactive peptide sequences, to evaluate the potential bioactivity of peptides in enzymatic hydrolysis, guiding enzymatic hydrolysis [11]. However, the intrinsic complexity of hydrolysates, coupled with the broad specificity of commercial enzymes, often results in a disparity between simulated forecasts and actual experimental outcomes [12].

For this purpose, two-dimensional liquid chromatography-mass spectrometry (2D LC-MS) was employed, recognized for its superior peptide analytical capabilities [13]. In this separation method, two chromatographic columns are interconnected by valve-switching technology, ensuring that each compound undergoes exhaustive chromatographic separation, resulting in unparalleled resolution [14]. A robust analytical platform has been developed by our team, specifically designed for the assessment of food protein enzymatic hydrolysates, meeting the predefined objectives [13].

Elastin was selected as the model substrate to investigate its elastase inhibitory activity during enzymatic hydrolysis. In previous research conducted by our team, the potential of bovine elastin hydrolysates to counteract skin photoaging was highlighted, with peptides such as GLPY and GV being observed to exhibit significant elastase inhibitory properties [15]. As indicated in the UniProt database, repetitive segments are found in bovine elastin, suggesting the potential for a high yield of specific bioactive peptides upon hydrolysis [16]. It is noteworthy that a substantial amount of elastin is found in bovine arteries, which are byproducts of the beef industry [17].

In this research, based on the new strategies discussed above, the release dynamics of amino acids and elastin peptides during enzymatic hydrolysis of elastin are sought to be elucidated, and the enzymatic cleavage paradigms under the given experimental conditions are to be inferred. Sophisticated separation and characterization techniques were employed for the delineation and profiling of peptides. An assessment is aimed to be made regarding the influence of high-concentration elastin peptides on the elastase inhibitory capacity of elastin hydrolysates. By deciphering the fluctuations in elastase inhibitory capacities and pinpointing pivotal peptide constituents, the foundation for the industrially viable production of potent elastase peptide-inhibitors from bovine arteries will be laid.

## 2. Results and Discussion

### 2.1. Hydrolysis of Elastin: A Process to Produce Elastase Inhibitory Peptides

#### 2.1.1. Changes in Protein Recovery and Degree of Hydrolysis (DH)

**Figure 1:**
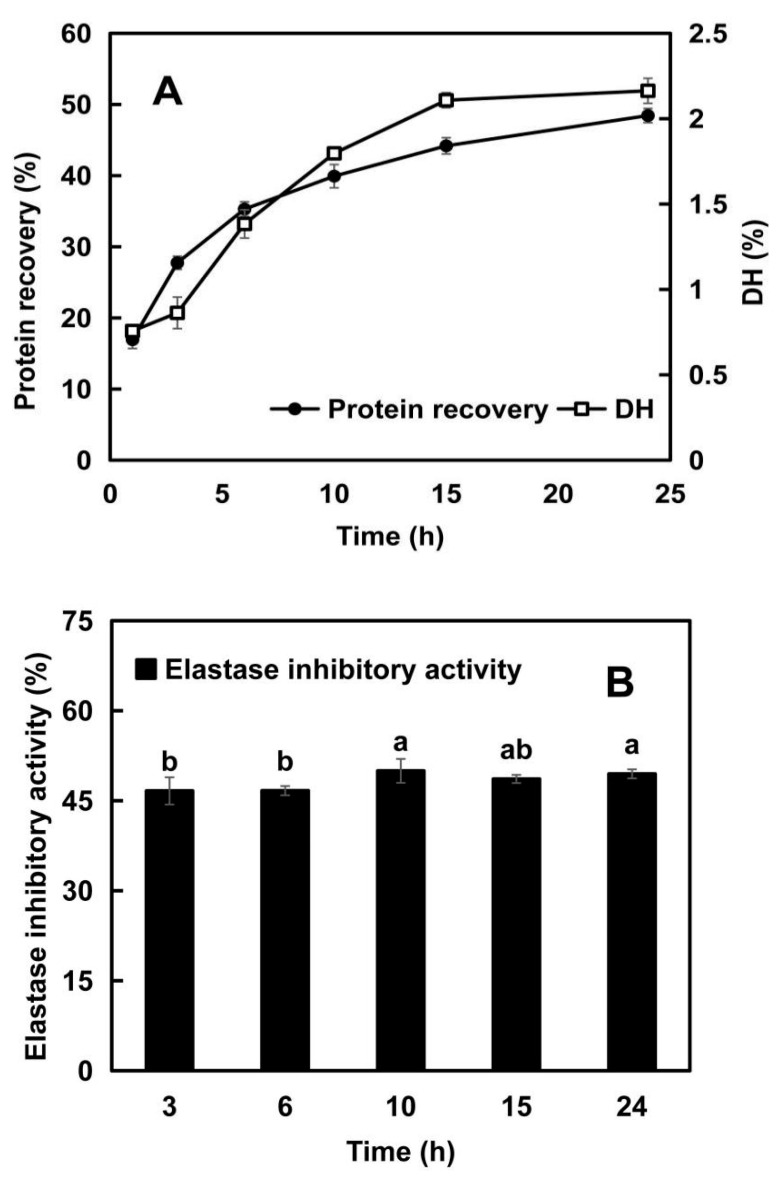
Enzymatic hydrolysis efficiency (**A**) and elastase inhibitory activity (**B**) of elastin hydrolysates obtained after different enzymatic hydrolysis times. In Panel (**A**), the left *Y*-axis represents the protein recovery (%), and the right *Y*-axis indicates the degree of hydrolysis (DH, %). In Panel (**B**), bars labeled with different letters indicate statistically significant differences (*p* < 0.05).

In the preliminary screening experiments, Alcalase^®^ 2.4L was selected as the food-grade protease with the highest enzymatic efficiency and elastase inhibitory activity. Alcalase^®^ 2.4L, synthesized by the Bacillus species, operates optimally under mildly alkaline conditions. As an endopeptidase-rich commercial protease formulation, it can effectively break many proteins down into smaller peptides and amino acids [18]. To investigate its hydrolysis efficiency, the protein recovery and DH of elastin hydrolysates obtained at different hydrolysis times by Alcalase^®^ 2.4L were evaluated. The former denotes the retained protein post-hydrolysis, the latter measures the extent to which protein is broken down into peptides and amino acids [7]. As shown in Figure 1A, the protein recovery increased rapidly with prolonging hydrolysis time (from 27.75% to 44.19%) and then tended to plateau (48.44% at 24 h). The change in DH showed a similar trend to protein recovery rate. However, the maximum hydrolysis yield and DH value were only 48.44% and 2.16%, indicating the difficulty for Alcalase^®^ 2.4L to extensively hydrolyze elastin. This could be due to elastin being an insoluble and highly crosslinked protein [19].

#### 2.1.2. Changes in Elastase Inhibitory Activity with the Hydrolysis Time

In general, large peptides accompanied by small peptides and free amino acids were released during the early stage of hydrolysis, after which the large peptides were further degraded, resulting in a decrease in the contents of larger peptides and an increase in the amounts of smaller peptides or amino acids [20]. Different elastin peptides have different elastase inhibitory activities [15]. Meanwhile, there is also synergistic enhancement between compounds. Therefore, samples with different compositions have different inhibitory activities [5]. Elastase inhibitory activity refers to the ability of certain peptides to inhibit the activity of elastase, an enzyme that breaks down elastin (elastin is a protein found in connective tissue that imparts elasticity to tissues) [15].

From Figure 1B, there is a slight increase in the elastase inhibitory activity of EHs with the extension of hydrolysis time, and then it reached a plateau around the 10th h, which did not agree completely with the trend of DH. Although the increased DH indicated more peptides released from the elastin, the elastase inhibitory capacity was mainly determined by how the components within EHs interacted with elastase. It also depended on the structure, size, and amino acid sequence of peptides. Wu et al. [21] put forward similar views when they discussed the association of DH with the angiotensin-converting-enzyme (ACE) inhibitory activity of sweet sorghum grain protein hydrolysates. Moreover, only small variations were detected in the elastase inhibitory activity (45.64–49.97%) for all the elastin hydrolysates obtained after different hydrolysis times (from EH3 to EH24) (Figure 1A), indicating only a slight influence of hydrolysis time on its elastase inhibitory capacity.

#### 2.1.3. Amino Acid Composition of Elastin Hydrolysates Obtained after Hydrolysis for Different Times

**Figure 2:**
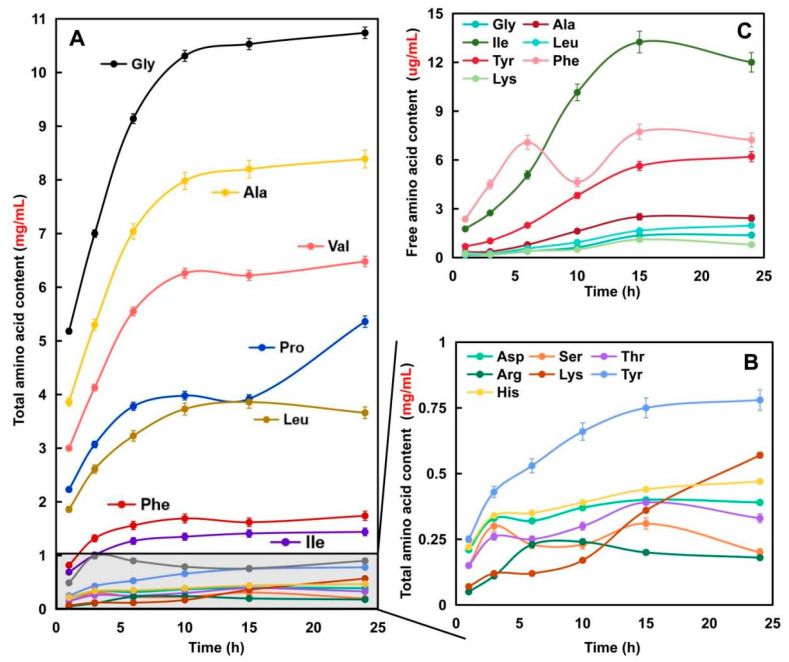
Analysis of total (**A**,**B**) and free (**C**) amino acid composition in elastin hydrolysates over various enzymatic hydrolysis times. Panel (**A**) presents the total amino acid content showing the concentration of all amino acids (mg/mL) in the hydrolysate. Panel (**B**) provides a detailed view of the less abundant amino acids from Panel (**A**), highlighting their content changes over time at an amplified scale (mg/mL) and Panel (**C**) illustrates the content of free amino acids (μg/mL) as they accumulate over time.

Figure 2A,B show the changing trends in total amino acid content of the samples. As illustrated, with prolonged enzymatic hydrolysis time, the content of each amino acid continued to increase and plateaued after 10 h. The total amino acid content increased from 19.22 to 41.64 mg/mL, where Gly accounted for over 25%, followed by Ala, Val, Pro, and Leu (cumulative proportion over 50%). The contents of other amino acids were negligible at less than 1 mg/mL. This is attributed to the nonpolar amino acid-rich characteristic of elastin (over 85%), with Gly accounting for around 1/3 [22]. Thus, the elastin hydrolysates exhibited a unique total amino acid composition. However, the Pro content increased by 36.95% at 24 h compared to 15 h, indicating substantial release of Pro or Pro-containing peptides in the later stage of enzymatic hydrolysis. Moreover, as shown in Figure 2C, the total free amino acid content in the samples was merely 5.80–33.23 μg/mL, accounting for only 0.03–0.084% of total amino acids. This demonstrates that the amino acids in elastin hydrolysates existed predominantly in polypeptide form. The detected free amino acids included only Gly, Ala, Ile, Leu, Tyr, Phe, and Lys, with Ile as the most abundant. Principal component analysis (PCA) was performed on the amino acid composition and protease inhibitory activity of the samples (Figure S2). The results showed poor dimensionality reduction, indicating that the differentiation and activity correlation analysis of the samples should focus primarily on peptide composition rather than amino acid composition.

#### 2.1.4. Peptide Fingerprints of Elastin Hydrolysates Obtained after Hydrolysis for Different Time

**Figure 3:**
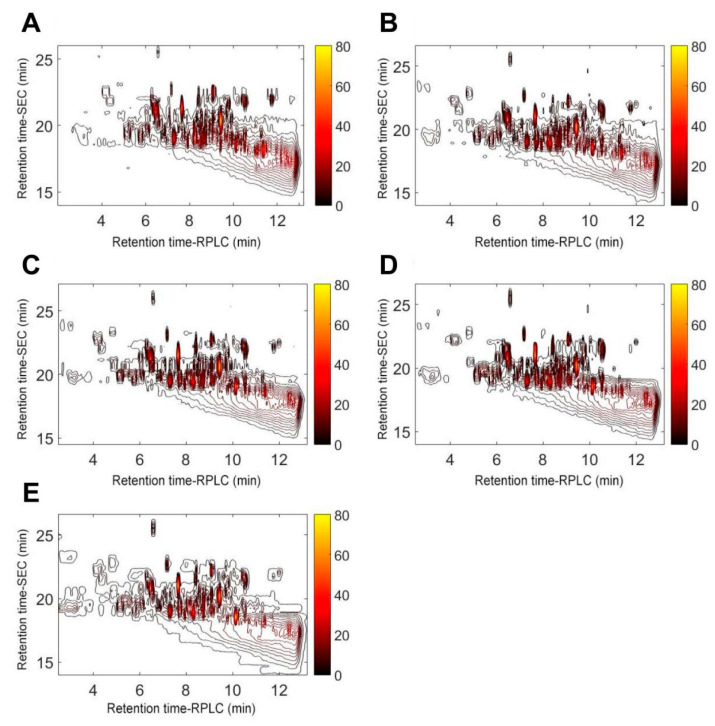
Stop-flow RPLC×SEC analysis of the elastin hydrolyzed by Alcalase^®^ 2.4L for 3 h (**A**), 6 h (**B**), 10 h (**C**), 15 h (**D**), and 24 h (**E**). The derived intensity-coded chromatograms are shown in each subfigure.

The peptide HPLC fingerprint profiles provide quantitative information about the peptide composition of hydrolysates. In this study, the peptide fingerprints (Figure 2) were successfully generated for EHs across different hydrolysis times using stop-flow SEC×RPLC. A larger number of peaks, ranging from 102 to 110, appeared in the chromatograms obtained using SEC×RPLC as compared with conventional RPLC [23]. The bovine elastin was fragmented into different peptides after hydrolysis over time. More peaks with higher intensities could be seen in the lower-right corner of Figure 3A (representing hydrolysis for 3 h), whereas these peaks were found in the upper-left corner of Figure 3E (representing hydrolysis for 24 h). Given that the first-dimensional (1D) SEC separation is mainly based on the molecular weight of analytes while the second-dimensional RPLC separation primarily relates to the polarity of analytes [10,13], one might conclude that the content of peptides with lower molecular weight and/or greater polarity increased with hydrolysis time.

According to Figure 3 and Supplementary File (Table S1), there were a total of 28 fragments with a relatively high content (the area percentage over 0.25%) with visible differences in the peak areas of F1–F28, suggesting that the contents of peptides in EHs varied during hydrolysis. In particular, five peaks that were initially eluted before the 19th min in SEC disappeared after a prolonged hydrolysis (hydrolysis time ≥ 15 h). This result indicates a breakdown of peptides into smaller molecular weights, which aligns with the observed results in the DH. Furthermore, 23 peaks with a relatively high intensity were found in all hydrolysates with different hydrolysis times, indicating their roughly similar peptide compositions, which might partially account for the similar elastase inhibitory activities of EHs with different hydrolysis times (Figure 1B).

**Figure 4:**
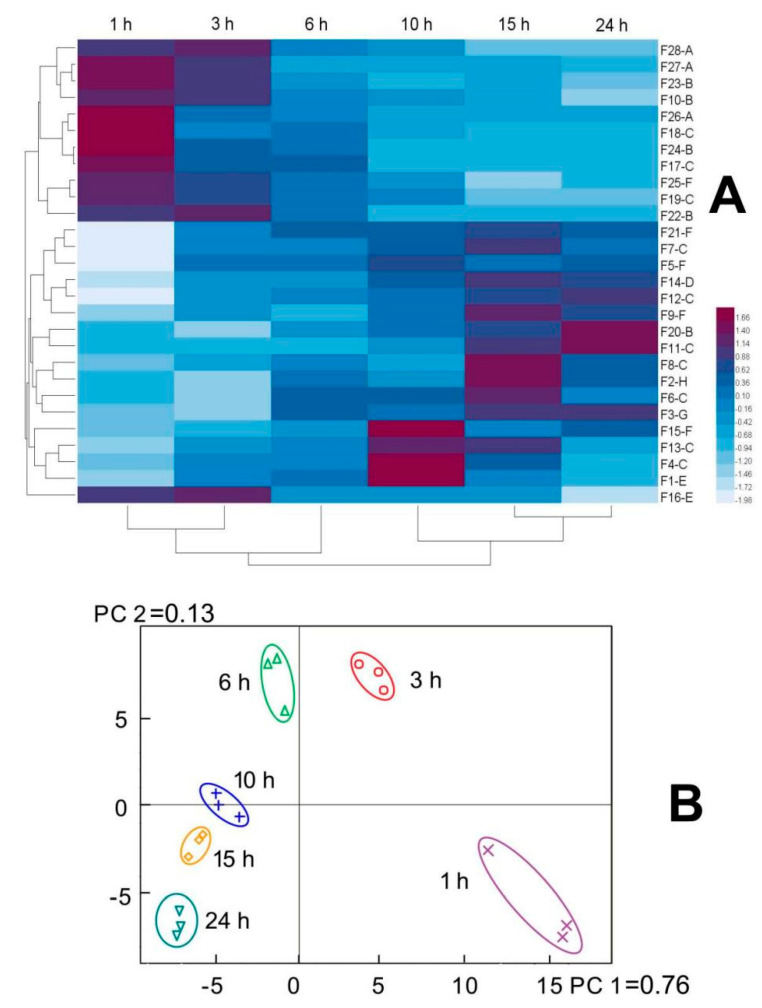
Analysis of characteristic peaks: heat map of characteristic peak area of elastin hydrolysates with different hydrolysis time (**A**); PCA plot to assess the variance of characteristic peaks of hydrolysates with different hydrolysis time (**B**).

To further elucidate the compositional changes of the samples during enzymatic hydrolysis, the peak areas of 28 characteristic peaks with high responses in Figure 3 were quantified (Figure 4A and Table S1). It should be noted that larger digits in the peak names indicate higher molecular weight, while letters later in the alphabet denote higher polarity (the schematic diagram presented as Figure S3). For instance, F28-A represents the largest molecular weight and lowest polarity, whereas F1-H indicates the smallest molecular weight and highest polarity. The results showed that components with higher molecular weight and lower polarity, such as F28-A and F10-B, exhibited decreasing peak areas with prolonged enzymatic hydrolysis time. In contrast, components with smaller molecular weight and/or higher polarity, including F8-C and F2-H, sequentially increased or first increased then decreased as hydrolysis time was extended. This further verifies that peptides with larger molecular weight and lower polarity were hydrolyzed into smaller molecular weight and/or higher polarity peptides over the course of enzymatic hydrolysis. Additionally, cluster analysis revealed that the compositions at 1 h and 3 h were similar but differed considerably from hydrolysates at other digestion times. When hydrolysis time exceeded 6 h, the sample compositions became increasingly analogous with increasing digestion time. The PCA results (Figure 4B) exhibited diminishing differences between samples obtained at different enzymatic hydrolysis times with increasing digestion duration. This further verifies the aforementioned findings.

### 2.2. Peptide Mapping of Elastin Hydrolysates

**Figure 5:**
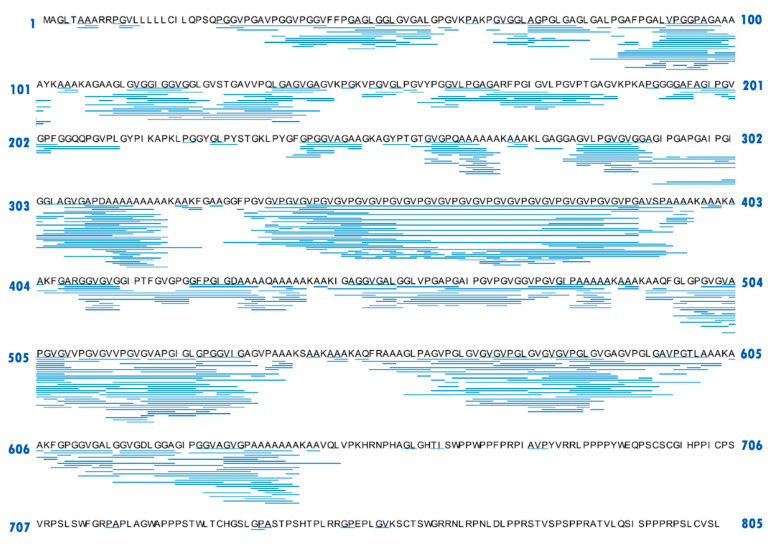
Comparative alignment of bovine elastin and the identified peptides (blue sticks) in the elastin hydrolysates generated by Alcalase^®^ 2.4L (10 h).

The correlation between the bioactivity and peptide sequence has been widely reported [24]. In this study, peptides in EH-10 (which exhibited the highest elastase inhibitory activity) were identified by UPLC-ESI-QTOF-MS/MS and comparative alignments of the identified peptides and bovine elastin were illustrated in Figure 5. As expected, quite a few long peptides were found in EHs, with the longest peptide (containing 33 amino acids) corresponding to the 294–326th amino acid at the N-terminal region of elastin. This result again suggested that elastin was difficult to be hydrolyzed deeply by Alcalase^®^ 2.4L, due to its crosslinked filamentous structure with many insoluble polymers [25]. Moreover, lots of peptide fragments were derived from the hydrophobic domains of the original elastin (e.g., from the 334th to 391th amino acid at the N-terminal region of elastin) (Figure 1). On the contrary, few peptides were identified in the terminal region, especially at the C-terminal region of elastin, which might be a result of the hydrolysis by Alcalase^®^ (an alkali enzyme) at pH 8.0. In addition to the reaction conditions, both the cleavage specificity and enzyme affinity towards proteins determine the profiles of the final hydrolysate [26]. Bounouala et al. [27] also reported different peptide products from the N-terminal and C-terminal regions: the peptides in 1–50th amino acid of the N-terminal region of the ovine casein could not be released by *Lactobacillus lactis*, whereas, both *L. lactis* and *Lb. brevis* were capable of releasing phenylalanine, leucine, or tyrosine at C-terminal in the hydrolysates.

On the basis of MS/MS results, peptides were identified and summarized in Table S2 with relevant information. As expected, Gly, Ala, Val, and Pro were frequently observed in the identified elastin peptides because of the characteristics of the amino acid composition of elastin. Our recent study [15] reported that peptides containing Gly, Ala, Pro, and Val residues exhibited extremely high elastase inhibitory property. Especially, elastase exhibited strong affinity to Gly, Ala, Pro, Val, and Leu, especially Gly and Ala [28]. Hence, the high levels of Gly, Ala, Val, and Pro residues in elastin peptides might be responsible for the observed high elastase inhibitory activity of EH-10.

### 2.3. Theoretical Content of the Identified Peptides from Elastin Hydrolysate

Currently, there are two main strategies for discovering key bio-active peptides: 1. Protein raw material—enzymatic hydrolysis—separation and purification—analysis and identification—synthesis and validation [6]; 2. Protein raw material—enzymatic hydrolysis—bioinformatics simulation and screening—synthesis and validation [29]. Combining the data in Figure 1, Figure 2 and Figure 3 and the specificity of elastin amino acid composition, our research discovered the third strategy: protein raw material—enzymatic hydrolysis—theoretical calculation of peptide content—synthesis and validation. Accordingly, this study examined the amino acid sequences with a high theoretical content in the elastin hydrolysates. Based on the amino acid sequence of the elastin obtained from the Uniprot database, abundant repetitive motifs could be found in bovine elastin (Figure 5), suggesting that these peptides with high contents could be released from elastin via proper hydrolysis and may have good bio-function contribution. The theoretical maximum repeating numbers of the peptides identified from EHs were calculated using MATLAB^®^ Soft (2019a) and listed in Table S2.

According to Table 1, 18 peptides with a theoretically high content (more than 5% (*w*/*w*) were presented. Sequences S1–S10 and S12 might be released from the 334th to 391th amino acid at the N-terminal region of elastin, and peptides S15–S18 could be obtained from several fragments such as the 263rd to 272nd and 637th to 646th amino acids at the N-terminal region of elastin. Specially, the dipeptide, GV, was repeated in elastin 80 times with the highest theoretical molar concentration (1106.24 μmol/g), followed by AA (which appeared 64 times). S2 (VGVPGVGVPGVGVPG) had the highest theoretical mass content (155.03 mg/g, equal to 15.50%). S1–S9 were generated from the same region of elastin and contained similar amino acid sequences, as did S15–S18 (Table 1). Accordingly, the peptides, S1, S9, S10, S12, S13, S14, S17, and S18 were chosen as the mark peptides, and the elastase inhibitory activities of these peptides were further investigated.

### 2.4. Release of the Peptide Markers during Hydrolysis of Elastin

The key elastase inhibitory peptides generated during the hydrolysis of elastin were analyzed using UPLC-ESI-MS/MS (Table 2). These peptides might be released directly upon the action of Alcalase^®^ 2.4L, or formed indirectly via the breakdown of the peptides with high molecular weights during hydrolysis. The sum content of peptide markers increased gradually with the hydrolysis time. The amounts of peptide markers in the elastin hydrolysates ranged from 0.23 to 70.90 μmol/g (on a dry basis). The content of S18 (AA) for each hydrolysis time (50.35~70.90 μmol/g, on a dry basis) was much higher than that of other peptides, whereas the contents of both S10 (VPGVG) and S12 (VPG) were less than 1 μmol/g (on a dry basis). These results suggested that Ala, Val, and Gly might be the preferred acting sites for Alcalase^®^ 2.4L in this experiment. Interestingly, although the terminal amino acids of S10 and S12 were Val and Gly, respectively, and they have smaller molecular weights than S1 or S9, it seemed to be difficult to obtain a higher yield of S10 and S12 during hydrolysis, as can be inferred from their low production levels (Table 2). Therefore, the effect of restriction enzyme cutting sites on the yield of a target peptide is limited. More work should be carried out to discuss the influence of other factors, such as the structure of precursors. A steady increase in the content of S13 (GV), S14, and S18 was detected during the whole hydrolysis, with an increasing rate from the 3rd h to 6th h in this order: S13 (62.27%) > S14 (25.26%) > S18 (11.84%). It indicated that S13, S14, and S18 were continuously released by Alcalase^®^ throughout the whole process of hydrolysis. In contrast, the content of S1 firstly increased and then decreased over hydrolysis with the peak content occurring at the 10th h, suggesting that S1 might be released from elastin at the initial hydrolysis stage, and then a portion of it was further broken down to smaller peptides.

### 2.5. Elastase Inhibitory Activity of the Marked Peptides in the Elastin Hydrolysates

**Figure 6:**
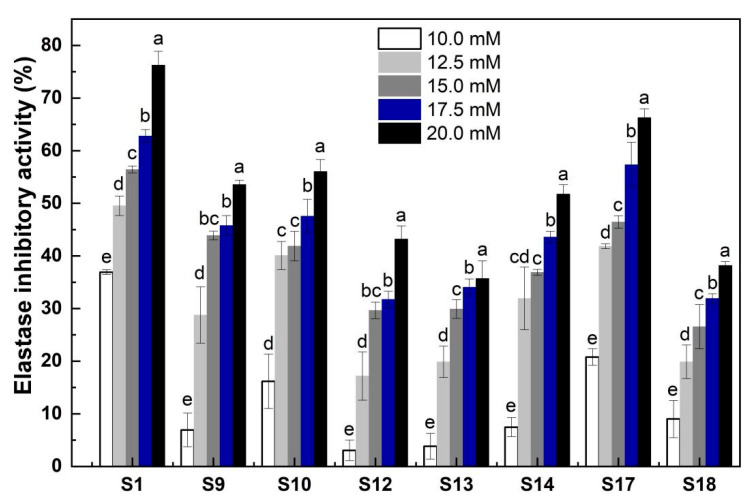
Elastase inhibitory activity (%) of the key peptides (S1, S9, S10, S12, S13, S14, S17, and S18) identified in the elastin hydrolysates at various concentrations (10.0 mM, 12.5 mM, 15.0 mM, 17.5 mM, and 20.0 mM). Bars with different lowercase letters (a, b, c, d, e) indicate significant differences (*p* < 0.05) within each sample at different concentrations.

A dose-dependent pattern was found for all the selected mark peptides in the concentration range of 10–20 mM: The elastase inhibitory activity was positively correlated with peptide concentration (Figure 6). At the same peptide concentration, S1 (VGVPGVGVPGVGVPGV) displayed the highest elastase inhibitory activity (e.g., 76.28% at the 20 mM) followed by S17 (AAA). At a lower concentration, like 10.0 mM, S13 (GV) and S12 (VPG) exhibited the lowest elastase inhibitory capacity, whilst at a high concentration, like 20.0 mM, S13 and S18 had the lowest inhibitory effect. At a particular concentration, some mark peptides exhibited similar levels of elastase inhibitory activity (Figure 6), such as S12, S13, and S18 and S9 and S14 at 12.5 mM; S9, S10, and S17 and S12, S13, and S18 at 15.0 mM, and S9, S10, and S14 and S12, S13, and S18 at 17.5 mM. These results suggested that the inhibition of elastase was affected by both the amount and type of peptide inhibitors. The inhibition of elastase proceeds when susceptible peptide bonds in the elastin are translocated to the enzyme active site. The outcome of the peptide inhibitor-elastase interactions might be associated with the peptide structure- and/or size-dependent entrance into the channel(s)/domain(s) of elastase as we found previously [15]. Among the peptides with a similar sequence (such as S12, S11, S9, and S1), their inhibitory activity seemed to be enhanced by an extension of peptide chain, probably due to the fact that peptides with a longer chain likely contained more active sites for interacting with the elastase. Since Ala at the second and third positions of C-terminal exhibited considerable affinity for elastase [28], the tripeptide, AAA, likely possessed a much higher elastase inhibitory activity than the dipeptide, AA. In comparison, the addition of EH-10 (15 mg/mL) could lead to an inhibition of 49.97% of elastase activity (Figure 1B). Therefore, the mark peptides examined were the effective components responsible for the elastase inhibitory activity of EH-10.

The mark peptides, except S1, showed higher elastase inhibition compared to the EHs. However, they accounted for just 4.34% of the total peptides in EHs, suggesting other factors influence the elastase inhibition of EHs. The actual amounts of peptides, their individual activities, and possible synergistic effects could also influence elastase inhibition. The mark peptides had dose-dependent inhibition (Figure 6). Thus, their lower levels in EH3 and EH6 may reduce inhibition. The slight decrease in EH 15 and 24 inhibition may relate to less S1 at 15 and 24 h hydrolysis (Table 2 and Figure 1A). Given their high inhibition and contents, S18 (AA) could strongly contribute to inhibition. Despite high inhibition, S12 and S10 had low contents (<0.5 mg/g), limiting their contribution. S13 had lower inhibition but 47.65–55.75 times more content than S12. Therefore, S13 may contribute more to the inhibition than S12. Previous studies show synergies among peptides in hydrolysates can significantly influence bioactivity [30]. Therefore, synergies among mark peptides may increase EHs inhibition. Differing peptide contents for F1–F28 in EHs of varying hydrolysis times (Table S1) likely explained the differences in total inhibition.

## 3. Materials and Methods

### 3.1. Materials and Chemicals

Fresh bovine arteries were purchased from Nansha market (Guangzhou, China). Elastase and N-Succinyle-Ala-Ala-Ala-pNA were purchased from Sigma-Aldrich (Shanghai, China). Alcalase^®^ 2.4L (200,000 U/mL) was obtained from Novozymes (Bagsvaerd, Denmark). All the solvents for chromatographic analyses were of HPLC grade. Other reagents were of analytical grade.

### 3.2. Isolation of Elastin from Bovine Arteries

The fresh bovine arteries were minced using AUX AUX-J312 meat grinder (ISUN electric appliance Co., Ltd., Foshan, China) after cleaning and washing, and then lyophilized using a freeze-dryer (R2L-100KPS, Kyowa Vacuum Engineering, Tokyo, Japan), and crushed into powder. One hundred grams of the lyophilized artery powder was dispersed in 1 L NaCl aqueous solution (1 M) and stirred at 40 °C for 4 h. The mixture was centrifuged at 12,000× *g* for 10 min at 20 °C using a CR22N refrigerated centrifuge (Hitachi Co., Ltd., Tokyo, Japan). Then, the recovered residue was further dispersed in 1 L NaOH aqueous solution (0.1 M) and stirred for 1 h in a boiling-water bath to remove fats and non-elastin proteins. After centrifugation at 12,000× *g* for 10 min at 20 °C, the resultant residue (isolated elastin) was collected, washed with demineralized water to neutrality, lyophilized, and stored at −18 °C until use.

The purity of isolated elastin was evaluated using sodium dodecyl sulphate-polyacrylamide gel electrophoresis (SDS-PAGE) according to the method of reported reference [19]: The isolated elastin samples were incubated at 95 °C for 15 min under reducing conditions (5% (*v*/*v*) 2-mercaptoethanol), and analyzed with a 10% (*w*/*v*) gel by using Coomassie brilliant blue staining. No major contaminations with soluble proteins were observed in the isolated elastin (Figure S1).

### 3.3. Preparation of Elastin Hydrolysates (EHs)

The above-prepared elastin was dispersed in deionized water (elastin/water = 1:10, *w*/*v*), and the pH of the mixture was adjusted to 8.0 with 1 M NaOH before the addition of Alcalase^®^ 2.4L (2% *w*/*w*, on a dry protein mass basis). The resultant mixture was incubated at 55 °C for 3–24 h and then heated in a boiling water bath for 15 min (to inactivate the enzyme). The obtained hydrolysate was centrifuged at 8000× *g* for 20 min at 4 °C. The collected supernatant (elastin hydrolysates, EHs) was lyophilized and stored at −18 °C till use.

### 3.4. Protein Recovery and DH of the Elastin Hydrolysates

Protein recovery was evaluated using the Kjeldahl nitrogen analysis [31]. DH was determined using an OPA assay [32]. An aliquot (300 μL) of the OPA reagent was mixed with 40 μL of a test sample, water (as the control), or serine (0.97 mM, as a standard). After 2 min reaction, the resultant mixtures were measured spectrophotometrically at 340 nm using Varioskan Flash Spectral Scan Multimode Plate Reader (Thermo Fisher Scientific, Waltham, MA, USA).

### 3.5. Amino Acid Analysis

The amino acid profiles of all the samples were analyzed using the methods reported previously [33]. Specifically, the total amino acid composition, except for tryptophan, was determined using an A300 Amino Acid Analyzer (MembraPure, Hennigsdorf, Germany) after hydrolyzing the samples at 110 °C for 24 h with 6 M hydrochloric acid. The free amino acid composition was assessed after protein precipitation at 2–4 °C for 60 min with 15% sulfosalicylic acid.

### 3.6. Stop-Flow Size-Exclusion Chromatography (SEC) × Reversed Phase Liquid Chromatography (RPLC) for Peptide Separation

This paper uses a self-assembled two-dimensional liquid chromatography system. The stop-flow SEC×RPLC peptide separation was performed following the method of Xu et al. [10]. The TSK-GEL G2000 SWXL column (7.8 × 300 mm, LOT. 501Y; Tosoh Bioscience, Shanghai, China) was used as the first dimension column whilst a BEH C18 column (4.6 × 250 mm, Waters, Shanghai, China) was set up as the second dimension column. The mobile phase used for the SEC column consisted of water and acetonitrile (90:10, *v*/*v*) with 0.1% (*v*/*v*) TFA at a flow rate of 1 mL/min. For the second dimension C18 separation, a gradient elution was employed at a flow rate of 1.5 mL/min with the mobile phase consisting of (A) acetonitrile and (B) water containing 0.04% (*v*/*v*) TFA for 0–5 min (90% B), 5–20 min (90–40% B), 20–25 min (40–90% B), and 25–30 min (90% B isocratic). A 20-s slice was transferred at a flow rate of 0.30 mL/min from the first dimension to the second dimension analysis. The temperature for columns was set at 30 °C. The injection volume of sample solutions onto SEC column was 10 μL and the characteristic absorbance of amide peptide bond was monitored at 214 nm.

### 3.7. Peptide Analysis by UPLC-ESI-QTOF-MS/MS

An ACQUITY ultra-high performance liquid chromatography (UPLC, Waters Corporation, Milford, MA, USA) system, coupled with an IMPACT II quadrupole-time-of-flight (Q-TOF) mass spectrometer (Bruker Daltonics, Bremen, Germany), was applied for identifying the peptides derived from EHs. The mass spectrometer was equipped with an electrospray ionization (ESI) source which is suitable for elastin peptide analysis. The samples were firstly separated using an Xbridge^TM^ BEH C18 column (2.1 mm × 150 mm, 1.7 μm, Waters Corporation, USA), with mobile phase consisting of solvent A (water) and B (acetonitrile with 0.1% *v/v* formic acid) eluted at a flow rate of 0.2 mL/min in the following gradient: 0–2 min (isocratic 10% B), 2–6 min (10–50% B), 6–8 min (50–10% B), and 8–10 min (isocratic 10%). The eluate was detected by mass spectrometer in a positive ion mode and the mass range was set at *m*/*z* 50–3000 and *m*/*z* 50–2000 for MS and MS/MS procedures, respectively. The parameters for the ESI interface were as follows: 3.5 kV capillary voltage, 180 °C drying gas temperature, 6 L/min drying gas flow, and 1 bar ESI nebulizer pressure. The obtained MS and MS/MS spectra were interpreted using Mascot Distiller v2.4.2.0 software (Matrix Science, Inc., Boston, MA, USA; http://www.matrixscience.com, accessed on 13 December 2018) as well as Data Analysis (Bruker Daltonics, Bremen, Germany) to identify the sequences of elastin peptides. The analysis of peptides was finished by Mascot software using UniProt protein database (significance threshold: *p* < 0.05; FDR: 1.5%; tolerance: 0.005 Da). The sequences of peptides were settled using Data Analysis software (version 4.4) by manual de novo. For quantification, the targeted peptides were measured in the positive MRM mode using optimized tuning parameters, namely the signal intensity ratios of specific fragmented ions to the parental ions were within a reasonable range (for each peptide, at least one y ion and b ion were selected as specific fragment ions). The content of targeted peptides was calculated according to the calibration curves of corresponding standards (purified peptide), respectively.

### 3.8. Elastase Inhibitory Activity Measurements

The elastase inhibition assay was carried out following the method of Yoo et al. [34] with slight modifications. The reaction mixtures containing 50 μL of N-Succinyle-Ala-Ala-Ala-pNA (1 mmol/L), 50 μL of elastase (60 mU/mL), and 200 μL of the test sample were set up separately in individual wells of a 96-well plate. All the solutions for the reaction mixtures were prepared in a 50 mM Tris-HCl buffer at pH 8.0. The Tris-HCl buffer at the same concentration but in the absence of a test sample was used as the control. Each test sample or the control was analyzed in triplicate. The 96-well plate containing the reaction mixtures with/without a test sample was incubated at 25 °C for 5 min in the Varioskan Flash Spectral Scan Multimode Plate Reader, and the reaction was terminated by adding 10 μL of HCl (2 M). The absorbance of the reactive product (the released *p*-nitroaniline within 15 s) was measured at 410 nm. The elastase inhibition was evaluated through determining the inhibitory effect of the test sample on the elastase activity against the control.

Elastase inhibitory activity (%) = 1 − (A_s_ − A_s0_)/(A_c_ − A_c0_) × 100%

where A_c_ − A_c0_ is the change in absorbance at 410 nm for the control (A_c0_ and A_c_ are the absorbance values of the control before and after the reaction, respectively); A_s_-A_s0_ is the change in absorbance at 410 nm for the test sample (A_s0_ and A_s_ are the absorbance values of the test sample before and after the reaction, respectively).

### 3.9. Statistical Analysis

Data were presented as “mean ± standard deviation”. The obtained results were subjected to one-way analysis of variance (ANOVA). Duncan’s new multiple range test was performed to determine the significant difference among samples at the 95% confidence interval using SPSS 19.0 software (SPSS Inc., Chicago, IL, USA).

## 4. Conclusions

This research focused on elastin as the substrate model, subjected to various durations of Alcalase^®^ 2.4L enzymatic treatment to produce a series of hydrolysates endowed with elastase inhibition capabilities. The kinetics of amino acid and peptide liberation during hydrolysis were scrutinized, which is beneficial to ascertain the sequence and bio-functional contributions of key peptides. The inhibitory activity of the EHs against elastase increased gradually with the duration of hydrolysis and tended to stabilize after 10 h (49.97%), suggesting the difficulty of Alcalase^®^ 2.4L in hydrolyzing elastin and the potential for further enhancement of the raw material’s bioactivity. The enzymatic hydrolysates were characterized predominantly by peptides whose composition served as a key determinant in differentiating the products of enzymatic hydrolysis and influencing their subsequent elastase inhibition efficacy. Throughout the hydrolysis process, substantial peptides from the elastin’s hydrophobic domains were liberated, particularly between the 334th to 391st amino acids of the N-terminal region, with a scant number emerging from the C-terminal. Moreover, peptides of lower molecular mass and greater polarity accumulated as the hydrolysis progressed, predominantly deriving from larger hydrophobic peptide fragments. Despite the resemblance in peptide composition of the hydrolysates over different hydrolysis intervals, variances in peptide concentration were evident, influencing the elastase inhibition potency. Simulative analytics revealed 18 peptides within the hydrolysates exceeding the 5% threshold of theoretical release content, where 8 exhibited dose-responsive inhibition. Despite the actual release of these peptides being merely about 10% of their theoretically predicted quantities, and although the release pattern did not fully coincide with theoretical projections, the general tendency aligned well with expectations, and further empirical evidence demonstrated their robust elastase inhibitory activity, identifying them as key peptides. Intriguingly, the marker peptides demonstrated exponential inhibition enhancement at concentrations surpassing 10/12.5 mM/mL, hinting at a non-linear dose efficacy curve for these peptides. This underpinned the importance of ensuring ample liberation of marker peptides beyond effective threshold concentrations within the hydrolysis milieu. Marker peptides S13, S14, S17, and S18 were pinpointed as significant contributors to the hydrolysate’s bioactivity, owing to their pronounced inhibition capabilities and their substantial presence post-hydrolysis. The synergistic interplay among peptide fractions was non-trivial in enhancing the bioactivity of the hydrolysates, underscoring the criticality of maximizing the discharge of essential peptides and the strategic modulation of the peptide matrix. This paper successfully explored the feasibility and advantages of a third strategy in investigating the bioactivity of enzymatic hydrolysates and identifying key peptides. Future endeavors should systematically elucidate the enzymatic hydrolysis paradigms across diverse proteases on elastin, amplify the synergistic modalities among key peptides, and guide the bespoke engineering of enzymatic hydrolysis protocols, with the aim to fully harness the innate protein to produce elastin hydrolysates with heightened bioactivity.

## Figures and Tables

**Table 1:** Peptide sequences with a high theoretical content (>5%) in the elastin hydrolysates.

Serial Number	Sequence	Frequency	Molecular Weight	Content (on a Dry Basis)	
				(mg/g)	(μmol/g)
S1	VGVPGVGVPGVGVPGV	8	1344.7765	148.76	110.62
S2	VGVPGVGVPGVGVPG	9	1245.7081	155.03	124.45
S3	GVPGVGVPGVGVPGV	8	1245.7081	137.81	110.62
S4	GVPGVGVPGVGVPG	9	1146.6397	142.70	124.45
S5	VPGVGVPGVGVPG	9	1090.6212	135.73	124.45
S6	PGVGVPGVGVPG	10	990.5498	136.97	138.28
S7	VGVPGVGVPGV	9	935.5440	116.43	124.45
S8	GVPGVGVPG	10	737.4072	101.97	138.28
S9	VPGVGVPG	10	680.3857	94.08	138.28
S10	VPGVG	15	427.2431	88.62	207.42
S11	GVGV	21	330.1903	95.88	290.39
S12	VPG	26	271.1532	97.49	359.53
S13	GV	80	174.1000	192.60	1106.24
S14	PG	60	172.0848	142.78	829.68
S15	AAAAA	12	373.1961	61.93	165.94
S16	AAAA	20	302.1590	83.57	276.56
S17	AAA	37	231.1219	118.25	511.64
S18	AA	64	160.0847	141.67	884.99

**Table 2:** The changes in the contents of the key peptides during hydrolysis. Numbers within the same row marked with different letters indicate statistically significant differences (*p* < 0.05).

Serial Number	Sequence	Content (μmol/g, on a Dry Basis) as a Function of Hydrolysis Time				
		3 h	6 h	10 h	15 h	24 h
S1	VGVPGVGVPGVGVPGV	6.95 ± 0.03 ^a^	8.26 ± 0.14 ^d^	8.65 ± 0.19 ^e^	8.02 ± 0.03 ^c^	7.33 ± 0.04 ^b^
S9	VPGVGVPG	3.67 ± 0.04 ^a^	5.07 ± 0.04 ^b^	6.06 ± 0.03 ^d^	5.99 ± 0.03 ^c^	6.23 ± 0.03 ^e^
S10	VPGVG	0.23 ± 0.02 ^a^	0.38 ± 0.02 ^b^	0.66 ± 0.01 ^c^	0.72 ± 0.03 ^d^	0.95 ± 0.03 ^e^
S12	VPG	0.27 ± 0.02 ^a^	0.36 ± 0.01 ^b^	0.52 ± 0.02 ^d^	0.45 ± 0.01 ^c^	0.50 ± 0.02 ^d^
S13	GV	14.02 ± 2.03 ^a^	22.75 ± 1.92 ^b^	35.35 ± 2.74 ^c^	34.21 ± 3.08 ^c^	43.32 ± 0.17 ^d^
S14	PG	5.62 ± 0.31 ^a^	7.04 ± 0.45 ^b^	10.60 ± 0.48 ^c^	10.26 ± 0.69 ^c^	12.36 ± 0.19 ^d^
S17	AAA	35.76 ± 0.49 ^a^	37.74 ± 0.84 ^b^	36.76 ± 0.39 ^ab^	37.51 ± 1.40 ^b^	39.83 ± 0.68 ^c^
S18	AA	50.35 ± 2.28 ^a^	56.31 ± 1.36 ^b^	60.91 ± 1.95 ^c^	63.91 ±2.16 ^c^	70.90 ± 2.92 ^d^
SUM		116.85 ± 4.45 ^a^	137.90 ± 3.72 ^b^	159.51 ± 5.11 ^c^	161.07 ± 6.30 ^d^	181.42 ± 2.95 ^e^

## Data Availability

Data are contained within the article and Supplementary Materials.

## Supplementary Materials

The following supporting information can be downloaded at: https://www.mdpi.com/article/10.3390/molecules28227534/s1, Figure S1: SDS-PAGE analysis of purified elastin and original bovine arteries. The SDS-PAGE gel was loaded with 500 μg dry mass of isolated elastin (Lane A) and 500 μg dry mass of original bovine arteries (Lane B). Due to its insoluble polymeric structure, intact elastin does not enter the gel matrix. Only soluble proteins (such as collagen) and elastin breakdown products with lower molecular weights are able to migrate into the gel. This SDS-PAGE analysis demonstrates the purity of the isolated elastin sample compared to the original bovine artery tissue. Figure S2: PCA analysis of amino acid composition and elastase inhibitory activity of elastin hydrolysates. Figure S3: Schematic diagram of the name of characteristic peak assignments for elastin hydrolysates. Table S1: The changes in the area percentage of the fractions during the hydrolysis of elastin. Table S2: The frequency of occurrence of the sequences identified from the elastin hydrolysates.

## Abbreviations

Amino acids are commonly represented by abbreviations: tri-letter codes that signify their full names (for instance, ‘Ala’ signifies ‘alanine’), and single-letter codes that denote residues within peptide chains (such as ‘A’ representing an alanine residue).
