# Supplementary material for: Exploring the Release of Elastin Peptides Generated from Enzymatic Hydrolysis of Bovine Elastin via Peptide Mapping
Source: Molecules. 2023 Nov 10;28(22):7534. doi: 10.3390/molecules28227534 (PMC10673124; doi:10.3390/molecules28227534)
Supplement: Supplementary file 1 [file molecules-28-07534-s001.zip › molecules-2667216-supplementary.pdf]

Supplementary material

Exploring the Release of Elastin Peptides Generated from Enzymatic Hydrolysis of Bovine Elastin via Peptide Mapping

Jianan Zhang [1,2], Yang Liu [3,*], Liwen Jiang [3], Tiantian Zhao [4], Guowan Su [1,2] and Mouming Zhao [1,2,*]

[1] School of Food Science and Engineering, South China University of Technology, Guangzhou 510640, China; fez.jianan@foxmail.com (J.Z.); fegwsu@scut.edu.cn (G.S.)

[2] Guangdong Food Green Processing and Nutrition Regulation Technologies Research Center, Guangzhou 510650, China

[3] College of Food Science and Technology, Hunan Agricultural University, Changsha 410125, China; hnndjlw@163.com

[4] Sericulture & Agri-Food Research Institute, Guangdong Academy of Agricultural Sciences, Key Laboratory of Functional Foods, Ministry of Agriculture and Rural Affairs, Guangdong Key Laboratory of Agricultural Products Processing, Guangzhou 510610, China; fettzhao1989@163.com

* Correspondence: fs.ly@hunau.edu.cn (Y.L.); femmzhao@scut.edu.cn (M.Z.); Tel./Fax: +86-20-87113914 (M.Z.)

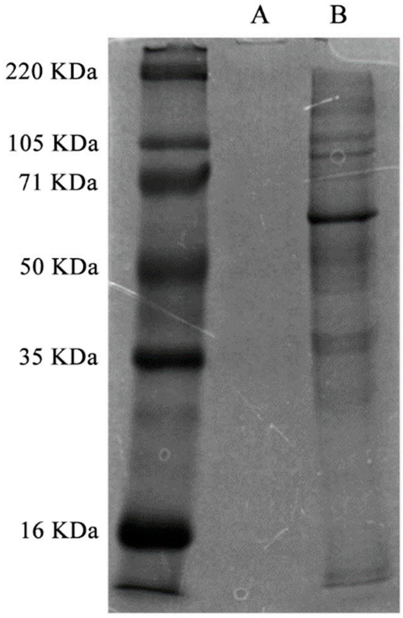

Figure S1. SDS-PAGE analysis of purified elastin and original bovine arteries. The SDS-PAGE gel was loaded with 500 µg dry mass of isolated elastin (Lane A) and 500 µg dry mass of original bovine arteries (Lane B). Due to its insoluble polymeric structure, intact elastin does not enter the gel matrix. Only soluble proteins (such as collagen) and elastin breakdown products with lower molecular weights are able to migrate into the gel. This SDS-PAGE analysis demonstrates the purity of the isolated elastin sample compared to the original bovine artery tissue.

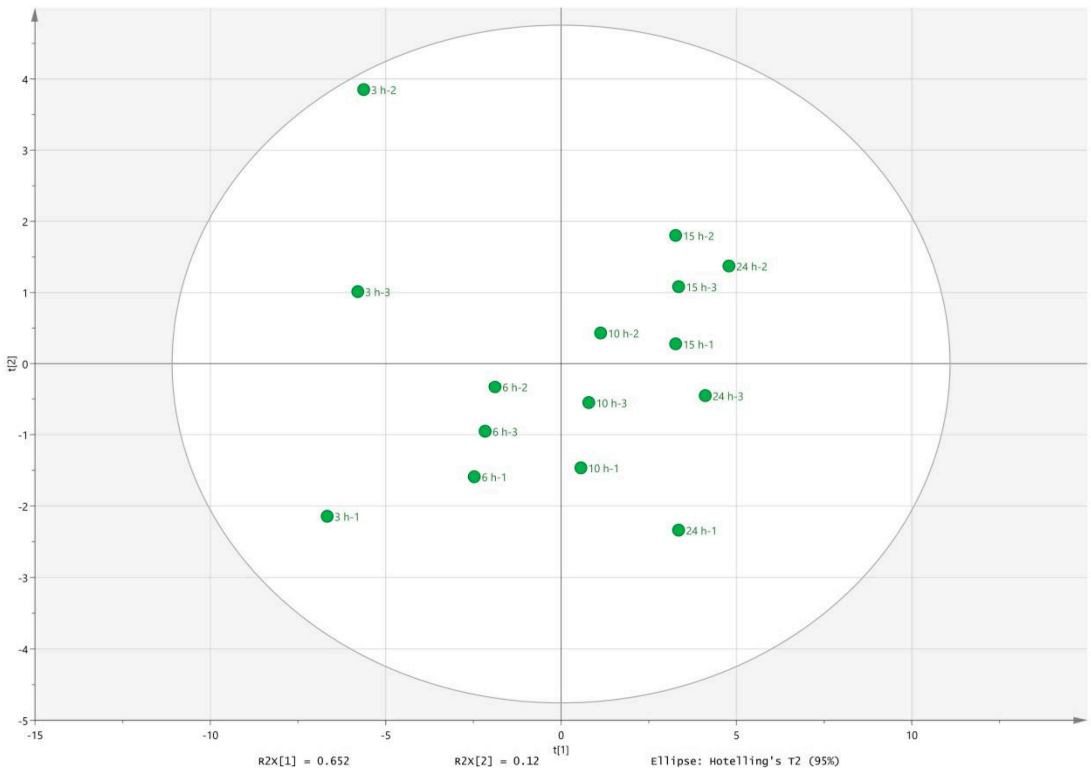

Figure S2. PCA analysis of amino acid composition and elastase inhibitory activity of elastin hydrolysates.

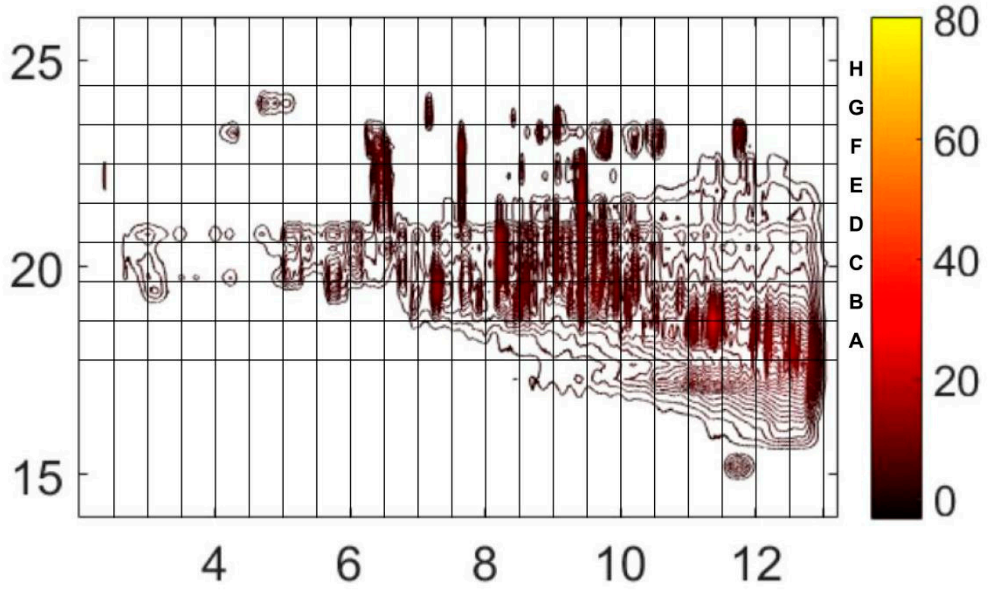

Figure S3. Schematic diagram of the name of characteristic peak assignments for elastin hydrolysates.

Table S1 The changes in the area percentage of the fractions during the hydrolysis of elastin.

Fraction	Area percentage (%) as a function of hydrolysis time				
	3 h	6 h	10 h	15 h	24 h
F1	3.47	2.02	1.65	1.08	1.06
F2	2.88	2.35	2.40	2.39	1.99
F3	2.84	1.92	1.23	1.15	1.13
F4	2.71	0.87	0.72	0.68	0.52
F5	2.69	1.65	1.23	1.31	1.03
F6	2.65	2.76	3.32	2.59	2.36
F7	2.45	2.85	2.42	3.95	3.21
F8	1.55	1.54	1.71	1.60	1.48
F9	1.35	1.40	1.62	1.44	1.51
F10	1.21	1.57	1.78	1.95	2.23
F11	1.07	0.70	0.50	0.41	0.13
F12	1.05	1.21	1.75	1.29	1.37
F13	1.01	1.20	1.31	1.49	1.59
F14	0.93	1.10	1.09	1.19	1.07
F15	0.93	1.34	1.37	1.54	1.23
F16	0.91	0.87	1.12	1.62	1.74
F17	0.90	0.97	1.75	1.58	0.74
F18	0.83	0.76	<0.05	<0.05	<0.05
F19	0.82	0.75	<0.05	<0.05	<0.05
F20	0.82	0.61	0.47	<0.05	<0.05
F21	0.71	0.88	0.26	<0.05	<0.05
F22	0.66	0.48	<0.05	<0.05	<0.05
F23	0.56	0.56	0.64	0.69	0.60
F24	0.42	0.43	0.51	0.57	0.54
F25	0.37	0.29	0.24	0.08	0.16
F26	0.26	0.20	0.34	0.50	0.43
F27	0.11	0.24	0.22	0.27	0.27
F28	0.10	0.21	0.17	0.30	0.22

Table S2 The frequency of occurrence of the sequences identified from the elastin hydrolysates.

Peptide	Repeats	Peptide	Repeats	Peptide	Repeats
GV	80	GVGGI	2	GPGGVAGAA	1
AA	64	VVPG	2	GVGAPDAA	1
PG	60	VVPGV	2	LVPGGPAG	1
AAA	37	QF	2	TGAVVPQ	1
VPG	26	AF	2	AGAAGLGVG	1
GVGV	21	AGVPGLG	2	GVGGIGGVG	1
AAAA	20	IGAG	2	GARGGVGV	1
GL	19	FGPG	2	ARGGVGVG	1
VPGVG	15	GAF	2	ALVPGGPA	1
GP	13	VVPGVG	2	VAPGIGLG	1
AAAAA	12	GAGI	2	VVPQLGA	1
VPGVGVPG	10	GAGL	2	VGPQAAAA	1
GVPGVGVPG	10	GPGGVAG	1	GVGPAAAAA	1
PGVGVPGVGVPG	10	GVGVGGA	1	GPGGVIGAG	1
VGVPGVGVPGV	9	GGVAGVG	1	GAVPGTLA	1
VPGVGVPGVGVPG	9	GVGGLAG	1	GIGGVGGLG	1
GVPGVGVPGVGVPG	9	PAAAAAA	1	GAFAGIPG	1
VGVPGVGVPGVGVPG	9	GVGPAAA	1	VPGGPAGAA	1
PA	9	AGPGLGA	1	VPGAVSPA	1
GVPGVGVPGVGVPGV	8	GAGLGGL	1	GVLPGVGV	1
VGVPGVGVPGVGVPGV	8	GIAGVGA	1	VLPGVGVG	1
PGGV	8	AGGVGAL	1	GVGDLGGAG	1
GAGV	6	LGAGVGA	1	GGVGDLGGA	1
AL	5	PGGVAGV	1	VPGVGIPA	1

GAL	5	GPGGVIG	1	AGPGLGAGL	1
PAAA	4	GVLPGAG	1	GVGVAPGVG	1
GVL	4	GGLVPGA	1	ARFPGIG	1
GGL	4	APDAAAA	1	GVGAGVPGL	1
VGP	4	AVSPAAA	1	PGLGVGAGV	1
VGVG	4	VAPGVGV	1	RAAAGLPA	1
GVGVG	4	PGGVAGAA	1	PDAAAAAAA	1
GVPGLGV	3	GVGPQAA	1	GVGAPDAAA	1
AQ	3	AGPGLGAG	1	GVGGIGGVGG	1
GVGAL	3	LGAGVGAG	1	AGAAGLGVGG	1
AKF	3	QLGAGVG	1	QLGAGVGAG	1
KF	3	LVPGAPG	1	GARGGVGVG	1
LGAG	3	LVPGGPA	1	GFPGIGDA	1
VGAL	3	PAGVPGL	1	LVPGGPAGA	1
AVP	3	PAAAAAAA	1	AGVPGLGAV	1
GVGALG	3	GVGPAAAA	1	GVGPQAAAA	1
GVGVPGL	2	VGPQAAA	1	GIPGVGPF	1
GVGVGV	2	GTGVGPQ	1	RFPGIGV	1
VPGLGVGVG	2	VGGIGGVG	1	GPAGAAAAY	1
GVPGLGVGV	2	GVGGIGGV	1	GVPGAVSPA	1
GVGVGVPGL	2	VPGGPAGA	1	GVLPGVGVG	1
PGVGVVPGV	2	VAPGIGL	1	AVVPQLGA	1
GVGVGVPGLG	2	PGLGVGAG	1	GVGPAAAAAA	1
GVGVPGLGVG	2	GVAGVGPA	1	GGAGIPGGVA	1
PGLGVGVGVPG	2	GPGGVIGA	1	GIAGVGAPD	1
GLGVGVGVPGL	2	GIGGVGGL	1	GVPGLGAVP	1
PGVGVVPGVGV	2	GLGVGGIG	1	VPGGPAGAAA	1

GVGVGVPGLGVG	2	GAFPGAL	1	AGPGLGAGLG	1
GVPGLGVGVGVPGL	2	GIPAAAAA	1	AVPGTLAAA	1
PGLGVGVGVPGLGV	2	AGIPGGVA	1	FGPGGVGAL	1
AAQ	2	GVLPGAGA	1	GARFPGIG	1
VAPG	2	GGLGVGAL	1	GLPAGVPGL	1
GVGGL	2	GVGDLGGA	1	VGVAPGIGL	1
GLGVGAGVPG	1	GVGVPGLGVGAG	1	VVPGVGVAPGIGL	1
TGAVVPQL	1	APDAAAAAAAAA	1	GGAGIPGGVAGVGPA	1
VGGIGGVGGL	1	GVGAPDAAAAAA	1	PGGVAGVGPAAAAAA	1
AGARFPGI	1	ALVPGGPAGAAA	1	AGIPGAPGAIPGIG	1
GVLPGAGAR	1	GPGGVIGAGVPA	1	GVPGLGVGAGVPGL	1
PDAAAAAAAA	1	AGIPGGVAGVGP	1	GVGAPDAAAAAAAAA	1
APDAAAAAAA	1	GIPGGVAGVGPA	1	AGVGAPDAAAAAAAA	1
GVGAPDAAAA	1	GLGVGAGVPGLG	1	GVSTGAVVPQLGA	1
LVPGGPAGAA	1	PGGFPGIGDAA	1	PGVGVPGVGVPGAV	1
PGVGVAPGVG	1	GGVGVGGIPTF	1	VAPGIGLGPGGVIG	1
ALVPGGPAGA	1	GLGGLGVGALGP	1	GIPGGVAGVGPAAAA	1
VLPGVPTGA	1	VVPQLGAGVGA	1	GVGPGGFPGIGDAA	1
GVLPGVGVGG	1	GIAGVGAPDAAA	1	GVPGGVFFPGAGL	1
AGIPGVGPF	1	AAQFGLGPGVG	1	GIAGVGAPDAAAAAA	1
PGVGVAPGIG	1	GVPGVGIPAAAA	1	VVPGVGVAPGIGLG	1
VLPGVGVGGA	1	LGPGVGVAPGVG	1	GVVPGVGVAPGIGL	1
AGVLPGVGVG	1	APGAIPGVPGVG	1	GAGIPGAPGAIPGIG	1
VGVPGLGVGA	1	PGLGVGAGVPGL	1	PGAPGAIPGIGGIAG	1
VGPQAAAAAA	1	VAPGIGLGPGGV	1	VAPGVGVVPGVGVV	1
GVGPAAAAAAA	1	PGVGVAPGIGLG	1	GVAPGIGLGPGGVIG	1
AGVGPAAAAAA	1	FGGQQPGVPL	1	GVPGGVFFPGAGLG	1

QFGLGPGVG	1	STGAVVPQLGA	1	VAPGIGLGPGGVIGA	1
VAPGIGLGPG	1	PGIGVLPGVPT	1	GLGPGVGVAPGVGVV	1
GLGVGGIGGVG	1	PGVGVVPGVGVA	1	GAFPGALVPGGPAGA	1
AGAAGLGVGGI	1	VAPGVGVVPGVG	1	GVGPGGFPGIGDAAA	1
ARGGVGVGGI	1	GAGVLPGVGVGGA	1	VPGAPGAIPGVPGVG	1
AAAKAAVQL	1	GVGAPDAAAAAAA	1	GGVGDLGGAGIPGGVA	1
KIGAGGVGAL	1	VVPGVGVAPGIG	1	GVLPGAGARFPGIG	1
AGLPAGVPGL	1	GPGGVIGAGVPAA	1	GVAPGIGLGPGGVIGA	1
GVFFPGAGL	1	GVSTGAVVPQL	1	VAPGIGLGPGGVIGAG	1
GAVPGGVPGGV	1	PGALVPGGPAGAA	1	GVGGLGVSTGAVVPQ	1
GPGVGVAPGVG	1	PGVGVVPGVGVV	1	PGVGVAPGIGLGPGGV	1
PDAAAAAAAAA	1	VPGVGVAPGIGL	1	GIPGGVAGVGPAAAAAA	1
GVGAPDAAAAA	1	GLGPGVGVAPGVG	1	GAFPGALVPGGPAGAA	1
APDAAAAAAAA	1	GVPGLGAVPGTL	1	RFPGIGVLPGVPT	1
STGAVVPQL	1	GVGVGVPGLGVGA	1	GVGPGGFPGIGDAAAA	1
FRAAAGLPA	1	GIAGVGAPDAAAA	1	GGLVPGAPGAIPGVPG	1
GFPGIGDAAA	1	AVSPA	1	VAPGIG	1
ALVPGGPAGAA	1	AY	1	GVGL	1
GVLPGVGVGGA	1	GGVL	1	GVGI	1
GAGVLPGVGVG	1	GVGGLA	1	AFPGAL	1
GVGVPGLGVGA	1	GVGPQ	1	VYPG	1
AGLGGLGVGAL	1	GGVGGL	1	AGPGLG	1
GVAPGIGLGPG	1	GVAGVG	1	GYPT	1
GLGVGAGVPGL	1	GVIGAG	1	PGGPA	1
GIAGVGAPDAA	1	AFPGALVPGGPA	1	TL	1
GARGGVGVGGI	1	GIGGIAGVGAPDA	1	TI	1
GIPTFGVGPG	1	IAGVGAPDAAAAA	1	AAQF	1

KFGPGGVGAL	1	GVGVGVPGLGVGV	1	LGPGGV	1
PGVGVAPGVGV	1	GLGVGGIGGVGGLG	1	LGPGVG	1
APGVGVVPGVG	1	PGGVFFPGAGLG	1	GVGPA	1
TGAVVPQLGA	1	PGAPGAIPGIGGI	1	LGGA	1
GVGALGGVGDL	1	GIPGAPGAIPGIG	1	GGVGDLG	1
GVSTGAVVPQ	1	LGPGVGVAPGVGV	1	GIPGAPGAIPGIGGIA	1
PGAIPGVPGVG	1	GVGAPDAAAAAAAA	1	GGVGVGGIPTFGVGPG	1
PGAIPGIGGIA	1	PGVGVAPGIGLGP	1	PGVGVVPGVGVAPGIG	1
FFPGAGLGGL	1	GVVPGVGVVPGVG	1	GVGVPGLGVGVGVPGL	1
GPGGVGAL	1	PFGGQQPGVPL	1	GLPAGVPGLGVGVGVP	1
PDAAAAA	1	GVGALGGVGDLGGA	1	VVPGVGVAPGIGLGPG	1
AGVGPAAAAA	1	VAPGIGLGPGGVI	1	GGFPGVGVPGVGVPGV	1
LVPGGPAGAAA	1	GAFPGALVPGGPA	1	LVPGAPGAIPGVPGVG	1
VAPGVGVVPGV	1	GIAGVGAPDAAAAA	1	GPGVGVAPGVGVVPGVG	1
GVPGGVFFPGA	1	GVGALGGLVPGAPG	1	GVPGGVFFPGAGLGGL	1
GLGPGVG	1	GLGVGAGVPGLGAV	1	GLVPGAPGAIPGVPGVG	1
GGVGGLG	1	PGIGVLPGVPTGA	1	RFPGIGVLPGVPTGA	1
GQ	1	APGVG	1	GALPGAFPGALVPGGPA	1
GQQ	1	GAAGL	1	GGLVPGAPGAIPGVPGVG	1
PAGAA	1	GVGAPD	1	GVGVGVPGLGVGVGVPGL	1
AQAAA	1	GGVGDL	1	GVPGVGVPGVGVPGAVSP	1
AAQAA	1	GIGGVG	1	VPGVGVPGVGVPGAVSPA	1
GAPGAIPGIGGIAGVGAPDAAAAAAAAKAAKF	1	GFGPG	1	PGVGVPGVGVPGVGVPGVGVPGAVSP	1
GVPGVGVPGVGVPGVGVPGVGVPGAVS	1	AAGLG	1	VGVPGVGVPGVGVPGVGVPGAVSPAAA	1
AGVGP	1	GIGL	1	PGVGVPGVGVPGVGVPGV<br>GVPGVGVPGAVSP	1
GGLA	1	PGGVIG	1	GGGAF	1

GVPGGV	1	VAPGVG	1	TLA	1
GLPY	1	AAY	1	GAFPG	1
AGPGL	1	GGAF	1	VAGV	1
GVGPG	1				
